# Magnetoconductivity and Terahertz Response of a HgCdTe Epitaxial Layer

**DOI:** 10.3390/s18124341

**Published:** 2018-12-08

**Authors:** Dmitriy Yavorskiy, Krzysztof Karpierz, Michał Baj, Małgorzata M. Bąk, Nikolai N. Mikhailov, Sergey A. Dvoretsky, Vladimir I. Gavrilenko, Wojciech Knap, Frederic Teppe, Jerzy Łusakowski

**Affiliations:** 1 Faculty of Physics, University of Warsaw, Pasteura 5, 02-093 Warsaw, Poland; dmitriy.yavorskiy@fuw.edu.pl (D.Y.); krzysztof.karpierz@fuw.edu.pl (K.K.); michal.baj@fuw.edu.pl (M.B.); malgorzata.maria.bak@student.uw.edu.pl (M.M.B.); 2 A. V. Rzhanov Institute of Semiconductor Physics, Siberian Branch RAS, 630090 Novosibirsk, Russia; mikhailov@isp.nsc.ru (N.N.M.); dvor@isp.nsc.ru (S.A.D.); 3 Institute for Physics of Microstructures, RAS, GSP-105, 603950 N. Novgorod, Russia; gavr@ipm.sci-nnov.ru; 4 Center for Terahertz Research and Applications, Institute of High Pressure Physics PAS, Sokołowska 29/37, 01-142 Warsaw, Poland; wojciech.knap@gmail.com (W.K.); frederic.teppe@umontpellier.fr (F.T.); 5 Laboratoire Charles Coulomb, UMR CNRS 5221, 34095 Montpellier, France

**Keywords:** THz magnetospectroscopy, magnetoconductivity, HgCdTe

## Abstract

An epitaxial layer of HgCdTe—a THz detector—was studied in magnetotransmission, magnetoconductivity and magnetophotoconductivity experiments at cryogenic temperatures. In the optical measurements, monochromatic excitation with photon frequency ranging from 0.05 THz to 2.5 THz was used. We show a resonant response of the detector at magnetic fields as small as 10 mT with the width of the resonant line equal to about 5 mT. Application of a circular polarizer at 2.5 THz measurements allowed for confirming selection rules predicted by the theory of optical transitions in a narrow-gap semiconductor and to estimate the band-gap to be equal to about 4.5 meV. The magnetoconductivity tensor was determined as a function of magnetic field and temperature 2 K < *T* < 120 K and analysed with a standard one-carrier conductivity model and the mobility spectrum technique. The sample showed n-type conductivity at all temperatures. At temperatures above about 30 K, conductivity was found to be reasonably described by the one-carrier model. At lower temperatures, this description is not accurate. The algorithm of the spectrum of mobility applied to data measured below 30 K showed presence of three types of carriers which were tentatively interpreted as electrons, light holes and heavy holes. The mobility of electrons and light holes is of the order of 10${}^{6}$ cm${}^{2}$/Vs at the lowest temperatures. Magnetophotoconductivity experiments allowed for proposing a detector working at 2 K and 50 mT with a flat response between 0.05 THz and 2.5 THz.

## 1. Introduction

For the last three decades, THz technologies have been becoming more and more important in many areas of technique and science. Boosted by demands in security and medical diagnosis, solutions based on application of THz radiation have obtained their firm position in different types of spectroscopy, imaging and sensing in medicine, pharmacology, biology, material science, communication, physics or chemistry. This rapid development of applications in such diverse branches of science and technique have been made possible mainly due two reasons. The first one is development of a robust spectroscopy technique, a THz time-domain spectroscopy (THz-TDS) which has become a basic THz spectroscopic tool worldwide, both in technical as well as scientific applications. There are numerous advantages of THz-TDS spectrometers offered commercially by several high-tech companies: they are relatively cheap (or, rather, not extremely expensive), small, reliable, fast and consume relatively small energy. In addition, they offer an insight both in a continuous wave (cw) as well as time-resolved analysis of processes studied. This should be compared with rather large THz Fourier spectrometers (not even speaking about grating-based ones) which require extreme experimental skill to get reliable data. In THz-TDS systems, a spectrum spans, typically, a range from about 0.2 THz to about 3 THz, while the dynamics can be traced with a sub-ps resolution. The second reason comes from the way THz radiation interacts with matter and two items should be addressed at this point. One should realize that this interaction is very often rather weak, which enables THz radiation to penetrate a broad class of materials giving a possibility to picture the inner structure of an object studied. In addition, there is a broad spectrum of quantum excitations whose characteristic frequency falls into the THz band: molecular vibrations, phonons, excitations in superconductors, quantum wells and quantum dots, plasmons (with all their rich variety), intra-impurity transitions or the cyclotron resonance. On the one hand, these excitations are the object of basic scientific research; on the other hand, some of them are the basis for construction of THz detectors or sources.

A question could be asked about whether cw techniques are still important in THz science and applications. The answer is positive for many reasons. The THz-TDS technique comes with a feature which is perplexing in some circumstances: the object studied is always exposed to a broadband THz radiation while a strictly monochromatic one is required in many cases. Second, monochromatic cw sources are typically much more powerful than TDS sources and thus are more appropriate in some experiments. Third, monochromatic radiation allows for a more dedicated use of well-developed methods of waveguiding to control of intensity, polarization and amplification of radiation. Last but not least, cw THz radiation is used in spectroscopy experiments where the external magnetic field plays the role of a dispersive optical element and tunes material’s resonances to fit the radiation frequency. These reasons are strong enough to stimulate development of cw THz detectors.

There are numerous examples where THz detectors find their application. Brucherseifer et al. [1] used a TDS system to carry out transmission measurements on polynucleotides and showed an analysis of the complex refractive index of DNA molecules. Zangeneh-Nejad and Safian [2] proposed a resonant THz sensor for label-free analysis. The main part of the device is to be composed of a ring resonator made of Si${}_{3}$N${}_{4}$ which would be coupled to a graphene strip ring resonator. A high-level performance of the sensor is expected to stem from its very small volume and it was estimated that the sensitivity of the device could be about 30 times higher than that of a corresponding optical structure. In another publication [3], these authors extended their approach to another system, based on graphene and MoS${}_{2}$. Since these two materials belong to so-called two-dimensional materials and one can fabricate them in a form comprising a given number of layers, an influence of the composition on the device’s performance was studied too. In particular, it was shown that a hybrid composed of a bilayer graphene and a monolayer of MoS${}_{2}$ should give the highest value of the figure of merit.

Metamaterials and metasurfaces have become an important part of application of THz radiation in sensing and controlling propagation of THz beams [4]. For example, Ref. [5], a response of a metasurface composed of a lattice of asymmetric split ring resonators to external THz radiation, was studied at a frequency range 0.8–1.2 THz and a very sharp feature in reflection was found at about 0.875 THz. It appeared that the position of this structure was sensitive to a load of the resonator with a dielectric material. This leads to construction of a sensor which is capable of detecting very small amounts of dielectrics. A general problem with application of THz metamaterials’ resonators is their relatively small quality factor. Recently, however, it has been shown that working at a Fano resonance or a quadrupol resonance can increase the quality factor over typical values by a factor of a few [6].

One of the major problems in THz systems is a small conversion efficiency of incoming radiation into measured signal. On the emission side, in THz-TDS application, it is important to increase the emitted power. This can be done by increasing the power of an increasing the power of a femtosecond laser used [7]. One can also play with the design of an antenna whose shape can be optimized to enhance performance of a TDS emitter (and detector) by a factor of a few tens [8,9]. In these cases, a better performance of a device supplied with such an antenna is caused by an enhancement of THz field resulting from a particular, comb-like, shape of metallization. An enhancement can be also achieved in split-ring resonators and or in tunneling structures; these issues were discussed in a recent review paper [10]. On the other hand, one can also play with the material composition of the antenna [11].

The present paper refers to research which is inscribed into the development path of sensitive cryogenic cw THz detectors. The works on cryogenic THz detectors show their importance in THz technology. The reason justifying these efforts is rather obvious: the energy of quanta in the THz range are an order of magnitude smaller than of thermal excitations at room temperature so it seems natural to cool the detector down to cryogenic temperatures. In addition, working with THz radiation, one often has to overcome problems resulting from low intensity of radiation to be detected, so a quest for high sensitivity detectors has been always actual. Let us note that such detectors are at the moment the most sensitive THz detectors and are used, in particular, in space applications. Generally, their high sensitivity is related to a cryogenic working temperature which reduces the thermal noise. For example, bolometers implemented in the Herschel mission, working at 300 mK showed the detectivity of the order of 10${}^{10}$ V/W with NEP (noise equivalent power) of the order of 10${}^{-16}$ W/$\sqrt{Hz}$ [12].

There are many types of cryogenic THz detectors: indium antimonide hot electron bolometers [13,14,15], quantum well infrared detectors [16,17,18], superconducting bolometers [19,20,21] or doped germanium bolometers [22,23,24]. In the present paper, we consider a detector based on an epitaxial layer of HgCdTe. Early works on Hg${}_{1-x}$Cd${}_{x}$Te crystals date back to the 50-ties of the last century when this material was used as a detector of mid-infrared radiation [25]. The history of research on Hg${}_{1-x}$Cd${}_{x}$Te covers almost 60 years now and has been marked with hundreds of papers devoted to these mixed crystals. During the last several decades, many reviews have appeared concerning different aspects of research carried out on Hg${}_{1-x}$Cd${}_{x}$Te (see, e.g., [26,27] and references therein) which can serve as a reference for studies that have been carried out in recent years. This material plays a special role among infrared detectors [28] because of its extreme sensitivity for the radiation with the wavelength of a few μm. HgCdTe detectors are also used for longer wavelength operation, at the THz band. For instance, they can be often found as detectors accompanying THz Fourier spectrometers. Analysis of their physical properties is mainly based on the Kane model [29] and its extensions (see, e.g., [30]). As for the applications, these materials have been widely used as detectors of mid- and far—infrared radiation (for a recent review, see [31]).

**Figure 1:**
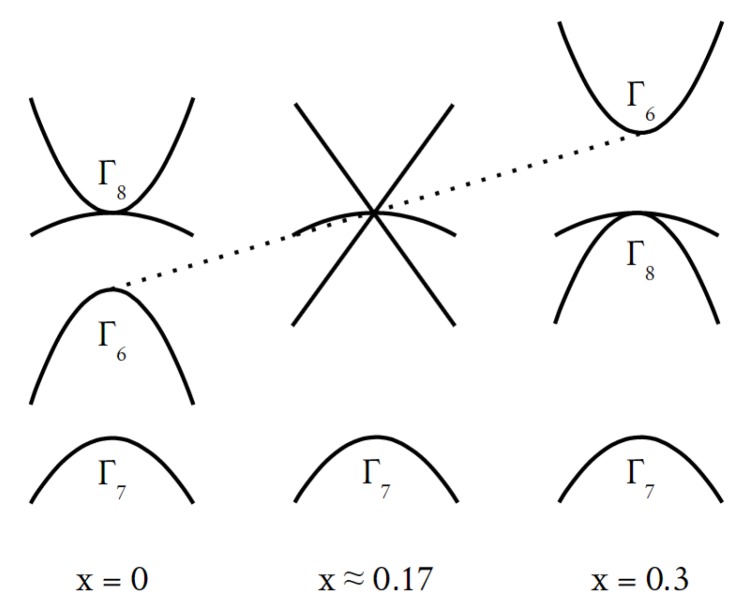
Evolution of bands in Hg${}_{1-x}$Cd${}_{x}$Te mixed crystals as a function of Cadmium content, *x*.

One of the most remarkable properties of Hg${}_{1-x}$Cd${}_{x}$Te is a dependence of its energy gap $E_{g}$ on Cadmium content *x*. A critical value of x≈0.17 marks a transition from a CdTe-like band structure with a “normal” ordering of bands at the Γ point of the Brillouin zone to a HgTe-like band structure when the bands are in an “inverted” order. In the CdTe-like ordering, a spin-orbit split-off band of light holes of a $\Gamma_{7}$ symmetry is positioned below a degenerate $\Gamma_{8}$ band of heavy and light holes and a conduction band of a $\Gamma_{6}$ symmetry. An HgTe-like ordering differs from the above by interchanging the $\Gamma_{6}$ and $\Gamma_{8}$ bands, which place $\Gamma_{7}$ and $\Gamma_{6}$ bands below that of $\Gamma_{8}$ symmetry. In this case, the energy gap is zero; one of the $\Gamma_{8}$ bands play the role of the valence band, while the other—that of the conduction band. This evolution of bands with changing *x* is schematically presented in Figure 1. At a critical value of *x*, the separation between the $\Gamma_{6}$ and $\Gamma_{8}$ decreases to zero and the dispersion of electrons and light holes becomes linear.

At this particular value of *x*, the band structure of Hg${}_{1-x}$Cd${}_{x}$Te is similar to that of graphene with an important difference resulting from the presence of the heavy holes band. This is why Hg${}_{1-x}$Cd${}_{x}$Te with x≈0.17 belongs to a “Dirac matter” which allows one to observe and study a number of fascinating phenomena resulting from the linear dispersion of bands. A recent review by Zawadzki [32] is strongly recommended as a guide through analogies between real Dirac (i.e., quantum and relativistic) particles and “Dirac particles” in solids [33,34].

Another fascinating field of interest which has been recently opened for experimental and theoretical studies is that of topological insulators [35]. An HgTe-CdTe system is perfectly well adapted for this kind of research because its energy gap can be tuned with Cadmium concentration, temperature or pressure [36]. This allows for crossing the transition between normal and topological insulator phase in an appropriately designed experiment. It should be stressed that both of these new directions of research have been made possible in Hg${}_{1-x}$Cd${}_{x}$Te thanks to availability of high-quality material grown by a molecular beam epitaxy.

These high-quality structures have been simultaneously tested as detectors of far-infrared radiation. One of the ideas is to look for THz detection with topologically protected surface states of a very high mobility. Tentatively, this would increase sensitivity of the detector because of reduction of scattering. Another reason is a general need to find a sensitive detector that could serve as a spectroscopic element to determine spectrum of radiation from a THz source. The idea behind this application is to profit from tuneability of a resonant transition with a magnetic field. This transition can be a cyclotron resonance [37], a magnetoplasmon resonance [38,39] or an intraimpurity transition [40]. An advantage of such a detector fabricated from Hg${}_{1-x}$Cd${}_{x}$Te comes from a small effective mass of electrons that leads to a fast change of the resonant energy with magnetic field for all these types of transitions. This offers a possibility to tune such a detector with a permanent magnet that would essentially reduce the cost and complexity of detector’s instrumentation.

The studies described in the present paper refer to complementary magnetotransport and magnetooptical experiments on a high-quality Hg${}_{1-x}$Cd${}_{x}$ epitaxial layer. The reason of carrying out these two types of measurements stems from the fact that the detection signal measured, i.e., a photocurrent, is influenced both by the optical response and direct current (dc) conductivity. In fact, a photocurrent signal is a result of two subsequent precesses. First, a THz photon is absorbed by an electron. The absorption can be resonant or non-resonant, but the final outcome is heating of the electron system. The second step is a change of dc conductivity related to an increased temperature of the electron system. For instance, in the case of a resonant absorption at the cyclotron resonance, electrons are transferred to the next higher Landau level (the optical response) where they contribute to the dc conductivity with a different mobility than in the initial state at the lower Landau level. A similar effect occurs in the case of a non-resonant absorption occurring within a single Landau level.

We consider in this paper a specific type of THz spectroscopy in which a monochromatic excitation is used and spectra are observed as a function of magnetic field. The magnetic field changes separation of quantum levels which take part in resonant transitions (like Landau levels or impurity levels) and it influences dc conductivity. One could think that, from the spectroscopy point of view, an influence of the magnetic field on dc conductivity is not important. This assumption is, however, not generally correct because it tacitly assumes that the width of spectral line (observed as a function of magnetic field) is much narrower than structures in conductivity generated by changes of *B*. There are, however, examples which contradict this conviction. For instance, in magnetoplasmon spectroscopy on samples of modulation doped GaAlAs/GaAs heterostructures with a very high electron mobility, one often has to carry out experiments at not too low temperatures (4 K rather than 2 K) because of strong Shubnikov—de Haas oscillations which dominate magnetoplasmon resonances [38,39]. As another example, in the case of CdTe/CdMgTe quantum structures uniformly doped with Iodine donors, magnetic field induced localization of electrons in an electrostatic fluctuating potential (i.e., a dependence of dc conductivity on magnetic field) is one of the factors which determine the shape of a photoconductive spectrum as a function of *B* [41]. In addition, the shape of response of an InSb magnetic-field-tuneable detector changes dramatically if one considers a photocurrent (i.e., a raw measured data) or photoconductivity (i.e., a recalculated photocurrent with taking into account detector’s magnetoresistance) [42]. This is why magnetic-field-tuneable detectors require characterization of their magnetotransport because structures related to optical transitions can interfere with or be modified by a magnetic field dependence of dc conductivity.

The content of this paper is composed of two parts. The first one deals with an analysis of the magnetoconductivity tensor $\hat{\sigma }$ measured as a function of temperature and magnetic field. We determined mobility of carriers describing $\hat{\sigma }\left(B\right)$ dependence with a model of one-carrier conductivity and with a spectrum of mobility technique. We showed that, in the whole temperature range 2 K–120 K, the sample behaves as an n-type semiconductor, but the description with the one-carrier model breaks at temperatures below about 30 K. The analysis with the spectrum of mobility technique indicates that more types of carriers are necessary to fit the experimental data at the lowest temperatures. These carriers are interpreted as electrons, light holes and heavy holes and the mobility of the first two is of the order of $10^{6}$ cm${}^{2}$/Vs below 10 K.

The second part of the paper describes transmission of a monochromatic radiation through the detector studied and photocurrent measurements. We used monochromatic THz beams with photon’s energy between about 0.05 THz and 2.5 THz. We show a resonant response at fields below 0.5 T. A polarization spectroscopy experiment carried out at 2.5 THz of the photons’ frequency allowed to establish the value of the energy gap equal to 4.5 meV. We also present photocurrent and photoconductivity of the detector at a very small magnetic field that shows that the detector working below about 0.05 T behaves as a non-resonant one in the whole range of photon’s frequency. A very strong dependence of the photoconductivity on a magnetic field around 0 T suggests an application in which the detector is switched on and off by modulation of the magnetic field. In addition, we show that, in the whole range of photon’s frequency and magnetic fields below 0.08 T, the photoconductivity (normalized to its value at *B* = 0) decreases with *B* with the same slope. This allows us to propose a detector with a flat response in a broad range of THz frequencies.

## 2. Materials and Methods

### The Sample and Experimental Procedures

**Figure 2:**
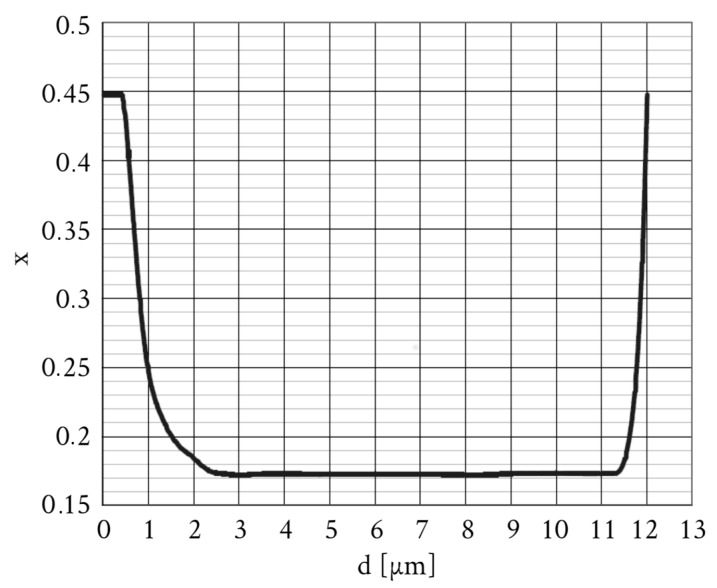
A content of Cadmium in the epitaxial layer studied as a function of the distance from the sample’s surface (at *d* = 0).

The sample studied was a molecular beam epitaxy-grown HgCdTe epitaxial layer. The active structure was grown on a semi-insulating GaAs with a CdTe buffer. The composition of the HgCdTe layer is shown in Figure 2. During measurements, the sample was kept in a variable temperature insert that allowed to change the temperature of the sample. The sample was positioned in the center of a superconducting coil and the magnetic field was directed along the layer’s growth axis. Conductivity measurements were carried out between 2 K and 120 K while THz spectroscopy at 2 K only because no clear signatures of THz-induced transitions were observed at higher temperatures.

The sample’s shape was a somewhat irregular rectangle with approximate dimensions of 4.5 mm × 6.5 mm. No lithography-defined structures were fabricated. The sample was supplied with four Indium soldered contacts. Gold wires of 25 μm of diameter were used to bond the sample to a multipin support. To avoid parasitic stress, the sample was glued at only one corner to the support. A hole in the support positioned just under the sample allowed for measuring the transmission of THz radiation that was registered with a bolometer (a thinned Alan-Bradley carbon resistor) glued to the back side of the support.

Magnetoconductivity measurements were carried out by the van der Pauw method with a dc current of 1 μA or 10 μA. To elliminate parasitic voltages, measurements were carried out for both directions of the current and both directions of the magnetic field, and measured voltages were combined according to the standard procedure. In addition, in order to reduce possible influence of sample’s inhomogeneity, measurements were repeated for different sets of contacts and resulting components of magnetoconductivity tensors were averaged. More experimental details can be found in [43].

Two sources of monochromatic THz radiation were used: a molecular (methanol) gas laser pumped with a CO${}_{2}$ laser and an electronic source based on frequency multipliers (a product of Virginia Diodes, Inc., Charlottesville, VA, USA). The THz beam was chopped with a mechanical chopper and a signal from the bolometer was registered as a function of magnetic field. Since the response time of the bolometer was rather long, the frequency of chopping was set to 13 Hz. A part of the THz beam was reflected from the chopper wheel and directed to a pyroelectric detector to monitor the beam intensity. Transmission data were next normalized to the readings of the pyroelectric detector.

Both conductivity and transmission measurements were carried out as a function of magnetic field which was swept with the speed of about 1 mT/s. In fact, the measurements were done as a function of the current flowing through the superconducting coil and then recalculated to the magnetic field with a known coil constant. A special care in measurements and data treatment was paid to a proper determination of the zero magnetic field. Due to residual magnetization of the sample’s environment, a zero current did not correspond to a zero magnetic field. This difference, which typically led to a magnetic field of 10 mT–20 mT at zero current, had to be taken into account in the case of low-temperature measurements where strong variations of the signal both in transport and spectroscopy measurements appeared at magnetic fields below about 50 mT. This problem was solved by a detailed registration of data with the magnetic field sweeping from −0.5 T to + 0.5 T and from 0.5 T to −0.5 T.

**Figure 3:**
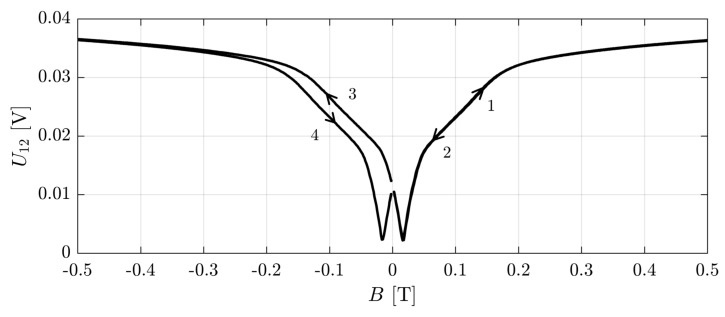
A voltage drop $U_{12}$ between two adjacent contacts (1 and 2) registered at the current of 1 μA supplied to the other pair of contacts (3 and 4). The horizontal scale shows the current flowing through the superconducting coil expressed as a coil magnetic field with the coil constant of 6.393 A/T.

An example of this procedure is presented in Figure 3 where a voltage drop $U_{12}$ between two adjacent contacts was measured at the current of 1 μA.

Measurements started at zero current which was then increased to give the field of about 0.5 T (arrow 1), then stopped and decreased (arrow 2), reversed direction (arrow 3) towards −0.5 T, stopped and returned to zero (arrow 4). One can see that this scan generated the curves which would be symmetric with respect to zero current if the part 1-2-3 were shifted to the left by about 20 mT and the part 4 were shifted to the right by the same amount. Thus, we could determine the position of the zero magnetic field with a precision better than 1 mT. This procedure was not so efficient at higher temperatures because the minimum of the signal was not so sharp. On the other hand, at higher temperatures, the signal did not show as sharp structures as at the lowest temperatures so the problem of shifting the data by about 10 mT was not relevant.

Measured values of resistances (described in detail in [43]) were transformed into components of the magnetoconductivity tensor $\hat{\sigma }\left(B\right)$. Since in the whole range of temperatures the conductivity was of the n-type, we started the analysis by applying a single-carrier model of conductivity tensors defined by Equation (1) with *N* = 1:

(1) $$\sigma_{xx}=\sum_{i=1}^{N}\frac{en_{i}\mu_{i}}{1+{\left(\mu_{i}B\right)}^{2}}\;,\;\sigma_{xy}=\sum_{i=1}^{N}\frac{en_{i}\mu_{i}^{2}B}{1+{\left(\mu_{i}B\right)}^{2}},$$

where $n_{i}$ and $\mu_{i}$ denote concentration and mobility of *i*’th type of carriers and *e* is the electron charge. We apply a convention according to which concentration and mobility is negative for electrons and positive for holes. As it will be shown below, this approach led to good results for temperatures higher than about 40 K. At lower temperatures, we have found signatures of a multi-carrier conductivity. Taking into account the band structure of the sample, one can expect the presence of electrons, light holes and heavy holes. This is why $\hat{\sigma }\left(B\right)$ at T<30 K was modelled with Equation (1) with *N* = 3. This, however, did not improve the quality of fits essentially or led to unphysical values of fitting parameters.

In such a case, we analysed $\hat{\sigma }\left(B\right)$ dependence with a technique called “spectrum of mobility” [44], this is a natural generalization of a multicarrier approach to description of a conductivity tensor which is based on replacement of expressions given by Equation (1) with integrals of the form:

(2) $$\sigma_{xx}=\int_{-\infty }^{\infty }d\mu \frac{s(\mu )}{1+{\left(\mu B\right)}^{2}}\;,\;\sigma_{xy}=\int_{-\infty }^{\infty }d\mu \frac{s(\mu )\mu B}{1+{\left(\mu B\right)}^{2}}.$$

The form of the integrand shows that the spectrum of mobility s(μ) is a conductivity per unit of mobility. The goal of the spectrum of mobility analysis is to determine the function s(μ). Let us note that, in the spectrum of mobility approach, one does not determine separately mobilities and concentrations of carriers involved in transport. The analysis rather gives information about contribution δσ to the total conductivity from carriers with mobility in the interval (μ,μ+δμ): δσ=s(μ)δμ.

The spectrum of mobility analysis was carried out in the frame of a maximum entropy approach. Details of this numerical procedure can be found in [45] and will not be repeated here. Before carrying out the analysis, we performed a series of numerical tests in order to find proper values of steering parameters of the spectrum of mobility procedure. These tests are described in Appendix A.

## 3. Results

### 3.1. Tensor of Magnetoconductivity and Mobility of Carriers in the Epitaxial Layer

**Figure 4:**
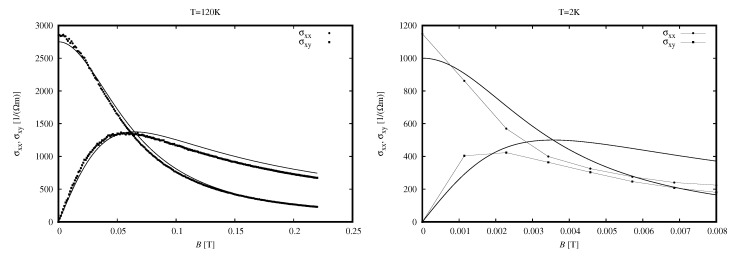
Components of the conductivity tensor at 120 K (**left**) and 2 K (**right**). Points: experimental results; smooth solid lines—fits of Equation (1) with *N* = 1. Note a different range of *B* in the two panels.

Magnetoconductivity tensor at 120 K and 2 K is shown in Figure 4 (at left and right, respectively). There is an important difference between these results. Data measured at 120 K is quite well described with a one-carrier model (Equation (1), *N* = 1). This is not surprising since, according to Equation (1) with *N* = 1, the plot of $\sigma_{xx}$ should cross the plot of $\sigma_{xy}$ at the maximum of the latter, and the height of the maximum should be two times smaller than $\sigma_{xx}\left(B=0\right)$. These conditions are quite well satisfied by the experimental data. In fact, such a degree of agreement between the *N* = 1 model and experimental data was found down to about 30 K.

At lower temperatures, discrepancy occurs and gradually increases with decreasing the temperature to finally reach the form presented in the right panel of Figure 4. One can see there that the crossing of experimental plots of $\sigma_{xx}$ and $\sigma_{xy}$ does not occur at predicted value of *B*, the maximum of $\sigma_{xy}$ is about three times smaller than $\sigma_{xy}\left(B=0\right)$ value, so the description with the *N* = 1 model is wrong. We checked that a description with a model with *N* = 3 did not lead to a satisfactory result, and this is why we analysed data with the spectrum of mobility technique.

**Figure 5:**
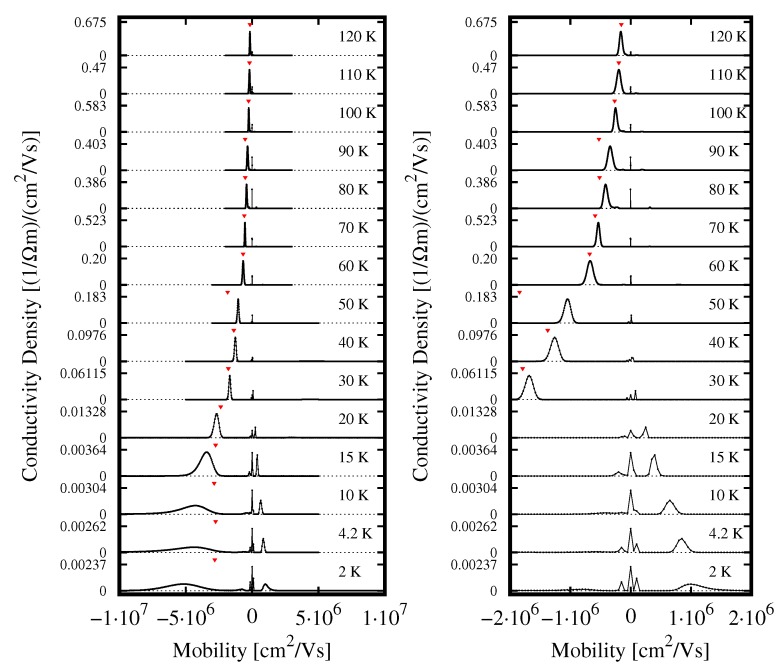
Evolution of the mobility spectrum as a function of temperature. Negative mobility corresponds to electrons, positive mobility to holes. The right panel shows an enlarged zero-centered part of the left panel. The spectra are shifted vertically for better presentation. Downward triangles mark results of fit of Equation (1) with *N* = 1.

Before processing experimental data, we performed detailed tests of the mobility spectrum software, as described in the Appendix A, and this step constituted an essential part of data analysis. Finally, based on results of the tests, we decided to apply the mobility spectrum software with 10${}^{7}$ iterations, the magnetic field range 0–0.5 T and the magnetic field step of 2 mT, being convinced that these parameters allow for obtaining reliable results. Nevertheless, one should be aware that the width of mobility peaks and their amplitude could change under variation of these parameters. Results of the spectrum of mobility analysis are presented in Figure 5; the figure also shows results of the *N* = 1 model (downward triangles).

One can see that, for temperatures down to about 30 K, the two methods of analysis give practically identical results. At lower *T*, a high-mobility component of conductivity related to holes appear. We interpret this component as caused by light holes. A contribution of light holes to the total conductivity is not big enough to change the overall type of conductivity—it is of n-type down to 2 K—but their presence can be clearly deduced from experimental data (deviations of $\sigma_{xx}\left(B\right)$ and $\sigma_{xy}\left(B\right)$ from the *N* = 1 model).

The right panel of Figure 5 shows that the evolution of the light hole peak starts only at temperature of about 30 K and a certain contribution to the conductivity comes from carriers of the mobility of the order of 10${}^{4}$ cm${}^{2}$/Vs, positive and negative (the peaks which are centered precisely at zero mobility are considered a numerical artefact; its signature is present in spectra at all temperatures). We would like to invoke two types of carriers that could be responsible for these two low-mobility peaks. The first one (leading to the positive mobility) could be heavy holes which must be present in the structure if the light holes are present—this is a direct consequence of the band structure shown in Figure 1. The second one (corresponding to the negative mobility) could be electrons moving in an impurity band. These hypotheses, however, should be tested by repeating measurements on samples with different levels of doping.

### 3.2. THz Spectroscopy of the Epitaxial Layer

**Figure 6:**
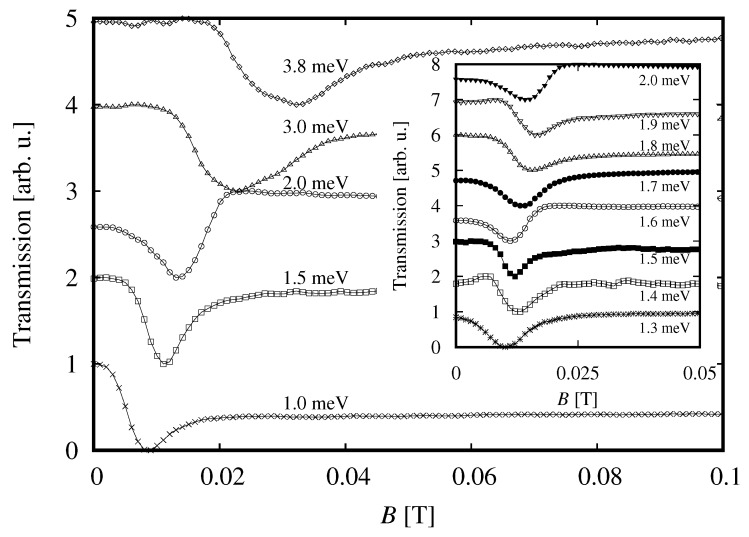
Transmission of a monochromatic radiation with photon energies indicated in the figure.

**Figure 7:**
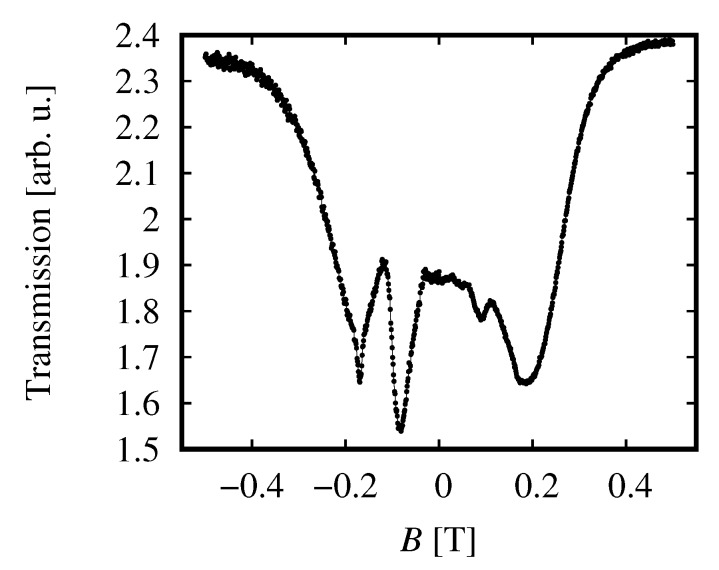
Transmission of 2.54 THz radiation in an experimental configuration with a circular polarizer.

Terahertz transmission measurements were carried out with monochromatic photon energies ranging from 1.0 meV to 3.8 meV (corresponding to the frequency band 0.25 THz–0.95 THz) and 10.16 meV (2.54 THz). Data of transmission of monochromatic radiation with lower photon frequencies are shown in Figure 6. The transmission spectrum shows a signature of a transition with the line width of about 10 mT which shifts to higher magnetic field with increasing the photon energy. At energies higher than about 3 meV, the line broadens and shows signatures of a splitting, which might evolve towards the splitting presented in Figure 7. The shape of the resonant feature presented in the inset to Figure 6 varies with the photon energy and we shall discuss this point in the next section.

In the case of transmission at 2.54 THz, a circular polarizer was placed close to the sample and the magnetic field was swept from negative to positive values. Then, $\sigma^{+}$ and $\sigma^{-}$ components of the transmitted signal were measured, as it is shown in Figure 7.

The spectrum shows lines at about ±0.1 T and at about ±0.2 T. The line at the lower *B* is strongly circularly polarized while the other one is not. Comparing this result with the selection rules of optical transitions presented in Ref. [34], we propose that the unpolarized transition is due to excitation from the heavy-hole band to the first Landau level. Assuming this interpretation, we can determine that the band-gap in the investigated sample is equal to about 4.5 meV. This value comes from a comparison of the position of the unpolarized line with calculations based on the Kane model presented in [34]. The polarized line is interpreted as a result of a transition between Landau levels in the conduction band, but more data (at other photon energy) are required to precisely indicate the levels involved in the transition.

**Figure 8:**
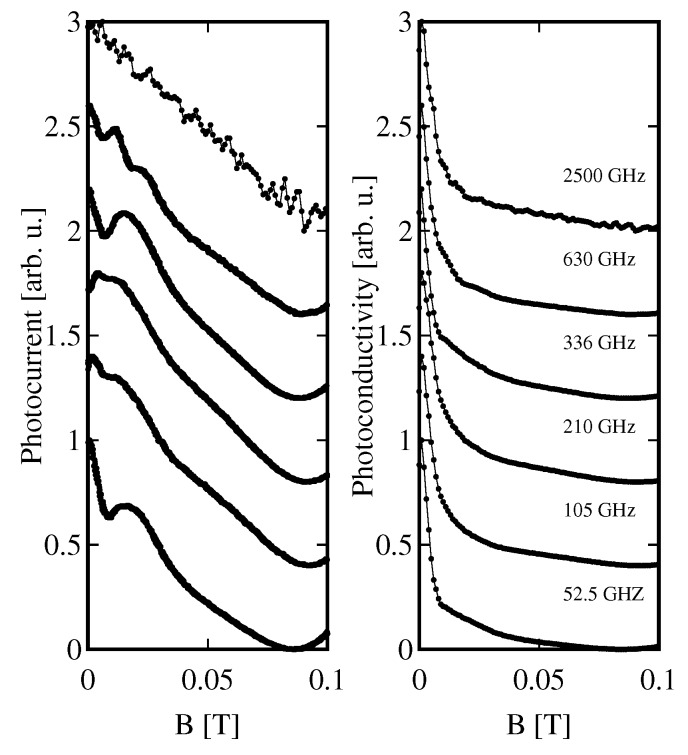
**Left**: photocurrent (normalized to the value at *B* = 0) measured at monochromatic excitation with radiation of frequency indicated in the right panel; **Right**: photoconductivity (normalized to the value at *B* = 0) at indicated frequencies. The curves in both panels are shifted vertically for better presentation.

Results of photocurrent measurements are shown in Figure 8, left panel. Taking into account the magnetoresistance of detector presented in Figure 3, we could calculate corresponding photoconductivity curves (Figure 8, right panel). Details of the recalculation procedure are described in [42]. As one can notice, a strong magnetoresistance at very low *B* changes drastically the shape of photocurrent on *B* dependence.

## 4. Discussion

### 4.1. Magnetoconductivity

An inspection of the evolution of the mobility spectrum in Figure 5 shows a systematic increase of electrons’ and light holes’ mobility up to values of a few times 10${}^{6}$ cm${}^{2}$/Vs at the lowest temperatures. In addition, a peak-like spectrum at the highest temperatures evolves into very broad peaks at the lowest *T*. These broad peaks probably reflect an in-plane sample’s inhomogeneity and also the fact that the sample is not uniform in the growth direction (see Figure 2).

One can easily notice that the strongest variations of the conductivity occur at very small magnetic fields. This concerns, in particular, the lowest temperatures, when a Hg${}_{1-x}$Cd${}_{x}$Te sample can be used as a THz detector. In a previous paragraph, we have shown that resonances in transmission for photon energy less than about 4 meV also occur at such small fields. This is a direct indication that application of such a layer as a spectroscopic resonant detector (i.e., tunable with magnetic field) must be done with a caution. If the detector is used in a conductive mode (measured signal is related to a dc conductivity), then it may happen that the optical response will be distorted by a strong σ(B) dependence. Such a situation was encountered in the case of an InSb detector when a proper analysis of measured photocurrent signal required taking into account magnetoresistance of the detector [42].

We notice that results of the one-carrier model differ at *T* = 50 K essentially from the mobility spectrum. Unfortunately, we cannot present an explanation of this discrepancy at the moment. A more detailed description of voltages measured during the magnetoconductivity experiment was described in Ref. [43], Figure 2. It was found that, just at 50 K, the Hall voltage measured showed rather strange symmetry with respect to the direction of *B*: it was neither antisymmetric (as it was at higher temperatures) nor quasi-symmetric (as it was at low temperatures), although it was antisymmetric with respect to direction of the current. Such a lack of symmetry could be a signature of particularly strong *B*—dependent parasitic voltages, but the questions remains open concerning both possible origin of such effects and why their influence only one—carrier model results. This question has to be answered by repeating measurements on a sample with a better defined geometry of the current lines.

### 4.2. THz Spectroscopy

Application of a circular polarizer enables to verify selection rules of transitions studied in a transmission experiment. A technical problem with THz polarizers is that fabrication of a monochromatic quarter wave plate for millimeter waves is very complicated (see, e.g., Ref. [46,47]) and such devices cannot be used in our experimental system. This is why polarization data were obtained only at 2.54 THz with the molecular laser as the source of radiation. This data can be considered as complementary to Fourier transform data presented in Ref. [34], where no polarizers were applied. As it was described above, the lines observed showed quite a different degree of polarization which essentially helped in identification of transitions to determine the band gap.

The shape of transmission spectra shown in Figure 6 resembles, in general, a combination of dispersive and absorptive dependencies. At the moment, we do not have a clear explanation of this behaviour, which would require development of an appropriate model of propagation of a THz beam in a layered structure, but we would like to suggest that this results from a complicated optical properties of the system studied. In the simplest approximation, we can model the sample as composed of a thin layer of Hg${}_{1-x}$Cd${}_{x}$Te, with a complex index of refraction resonantly dependent on magnetic field, positioned on a dielectric substrate of a non-resonant character. To describe an optical response of this system, one has to take into account reflections from both layers and absorption in Hg${}_{1-x}$Cd${}_{x}$Te. Obviously, interferences could also influence the optical response.

In addition, at the present stage of experiment, one cannot exclude factors other than those related to interferences and reflections, but still wavelength-dependent. For instance, one could think about a wavelength-dependent diffraction pattern of radiation at the exit aperture of the waveguide (next to the sample). This generates a bunch of rays and thus angles of incidence of radiation on the sample. Similarly, polarization of the radiation, which is linear at the source, changes during propagation through waveguides, and these changes can be wavelength-dependent. At present, we do not fully control these factors. However, repeating measurements many times, we have been convinced about a generally complex shape of the resonances presented in Figure 6.

Thinking about application of a Hg${}_{1-x}$Cd${}_{x}$Te layer as a detector, one could profit from results of the polarization experiment by noticing that application of a circular polarizer could make narrower the spectral response of a detector by eliminating signatures of some transitions from spectra. The ideal tool would be an achromatic circular polarizer which, in principle, is feasible [46,47]. Let us also note that a narrow spectral response seems to be found only at the lowest magnetic fields because, at *B* higher than about 0.03 T, the resonant line splits into a few components.

Let us note that the slope of the normalized photocurrent curves (Figure 6) are independent from a reasonable accuracy of the frequency of radiation spanning the range from about 50 GHz to 2500 GHz. This allows us to propose a detector which would measure the slope of the normalized photocurrent vs. *B* curve by a slight variation of the magnetic field at around 50 mT. Such a small field as well as its variations are easily achieved with classical small-power coils. Since the slope of the normalized photocurrent does not change with the frequency, an expected response of such a detector would also be flat in a broad range of frequency.

## 5. Conclusions

In conclusion, we carried out measurements of THz transmission, photocurrent and dc magnetoconductivity of a Hg${}_{1-x}$Cd${}_{x}$Te epitaxial layer as a function of temperature and magnetic field. We confirmed optical selection rules of interband transitions and showed a resonant transmission at magnetic fields of several mT. We also showed that, at the lowest temperatures (about 2 K) and small magnetic fields, there appear very strong changes of conductivity with *B*, which lead to big differences in the shape of photocurrent and photoconductivity spectra. Based on the shape on the normalized photocurrent measured in a broad range of THz frequency, we propose a detector whose response would be flat between 50 GHz and 2.5 THz.

## Figures and Tables

**Figure A1:**
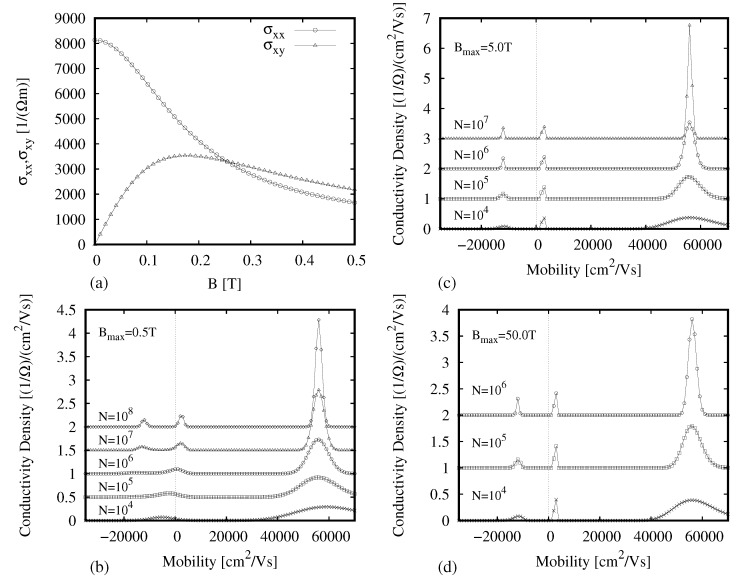
(**a**) Input data to test the numerical procedure of the mobility spectrum calculations. (**b**–**d**) Mobility spectrum calculated for different parameters *N*, δB and $B_{max}$, as described in the text.

## Appendix A

Since determination of spectrum of mobility is a complicated computational task, we present here results of some of tests which were carried out in order to establish proper values of steering parameters for the numerical calculus. In these test procedures, our aim was to determine mobilities of a system of three carriers with concentrations and mobilities given by $n_{1}=8.0\times 10^{15}$ cm${}^{-3}$, $\mu_{1}=5.6\times 10^{4}$ cm${}^{2}$/Vs, $n_{2}=14.0\times 10^{15}$ cm${}^{-3}$, $\mu_{2}=0.26\times 10^{4}$ cm${}^{2}$/Vs, $n_{3}=-2.0\times 10^{15}$ cm${}^{-3}$, $\mu_{3}=-1.2\times 10^{4}$ cm${}^{2}$/Vs.

Results of the test procedures are summarized in Figure A1. Figure A1a shows the input data (calculated, in fact, up to *B* = 50 T). Figure A1b–d shows how the results change when the input data are limited to $B_{max}$ equal to 0.5 T, 5 T and 50 T (b, c, d, respectively), and how sensitive the results to the number of iterations are, *N*. A general conclusion is that the width of mobility peaks shrink both with increasing *N* and $B_{max}$, but clearly one gains more in increasing *N* by an order of magnitude than *B*. Another test result (not shown) is that the mobility peaks shrink essentially when one decreases the increment δB of magnetic field in the data sets. The increment values down to 1 mT were tested without evidence of stabilization of the mobility peaks width. Obviously, for real data sets, experimental accuracy of measurements of the magnetic field as well as the value of $B_{max}$ put limits to optimization of these parameters.

Finally, taking all these considerations into account, and the time of acquisition required to register data with an appropriate precision in a reasonable range of magnetic fields, we decided to set $B_{max}$ = 0.5 T, δB = 1 mT and *N* = 10${}^{6}$.
